# Fatal Acute Disseminated Encephalomyelitis Post-COVID-19 Vaccination: A Rare Case Report

**DOI:** 10.7759/cureus.31810

**Published:** 2022-11-22

**Authors:** Shubham V Nimkar, Pallavi Yelne, Shilpa A Gaidhane, Sunil Kumar, Sourya Acharya, Rinkle R Gemnani

**Affiliations:** 1 Department of Medicine, Jawaharlal Nehru Medical College, Datta Meghe Institute of Medical Sciences (Deemed to be University), Wardha, IND; 2 Department of Epidemiology and Public Health, Jawaharlal Nehru Medical College, Datta Meghe Institute of Medical Sciences (Deemed to be University), Wardha, IND

**Keywords:** neurodeficits, myelitis, vaccination, diagnosis, encephalomyelitis, covid

## Abstract

Acute disseminated encephalomyelitis (ADEM) is a disease of the brain and spinal cord that is an immune-mediated inflammatory and demyelinating disorder, which is commonly preceded by an infection. Some reports have also shown the association of acute demyelination of the central nervous system (CNS) with vaccination. Primarily, the involvement of the white matter of the cerebral hemispheres, brain stem, and spinal cord is observed. Such lesions should be considered as the differentials of ADEM. We would like to report a case of a 77-year-old female who was of post-COVID-19 vaccination status and presented to us with altered sensorium with imaging revealing acute demyelination.

## Introduction

COVID-19 vaccination has been the breakthrough that the world needed to combat the SARS-CoV-2 viral infection. With many researchers working round the clock to create a perfect vaccine that is suitable to neutralize the maximum possible variants of the virus, there have been many vaccines that have entered the markets after passing through the various stages of trials, and the world population is being vaccinated with the available vaccines at a very fast pace. But with the concerns of efficacy and safety still being debatable among population groups, few cases of vaccine-induced myelitis have been reported. Traditionally seen as a complication of older vaccines such as hepatitis B, measles-mumps-rubella, rabies, and diphtheria-pertussis-tetanus [1], the recently available COVID-19 vaccinations have also had acute transverse myelitis-like events in a few patients who had taken the same.

## Case presentation

A 77-year-old female presented to our hospital with complaints of altered sensorium for four hours, aphasia for four hours, and loss of consciousness within one hour. The relatives have mentioned the presence of altered mental status for 15 days for which she was taken to an outside hospital where her routine blood investigations revealed the presence of hyponatremia and she was started on treatment for the same. Despite the initiation of treatment for hyponatremia, the patient had no improvement in the general condition. She had no history of fever or cough or cold. The patient was a known case of type II diabetes mellitus and systemic hypertension on regular medication. The patient had a history of infection with COVID-19 four months back and has also taken her first dose of COVID-19 vaccination 15 days back with AstraZeneca (ChAdOx1 nCov-19) vaccine.

In the emergency department, the Glasgow Coma Scale (GCS) of the patient was 5. In view of low GCS, the patient was intubated and put on a mechanical ventilator. On examination, the general condition of the patient was poor, she was febrile, her blood pressure (BP) was 150/90 mm Hg on the right arm in supine position, and her pulse rate was 124 beats per minute, regular and normal volume. Central nervous system (CNS) examination revealed the presence of neck stiffness. Cranial nerve examination was remarkable, the tone of the patient was increased in all four limbs, deep tendon reflexes were normal, and the plantars of the patient were mute bilaterally. CT screening of the brain of the patient done in the emergency department revealed no abnormality.

The patient was shifted to the ICU, and routine blood investigations were done. Her hemoglobin was 11.3 g/dl, her white blood cell count was 14700 cells/cu mm, and her serum sodium levels were 129 mEq/L. A lumbar puncture of the patient was done, which showed elevated cerebrospinal fluid (CSF) pressure and total white blood cell count of 47 cells/cu mm with 40% polymorphs and 60% lymphocytes. CSF glucose was 78 mg/dl with parallel plasma random blood sugar (RBS) of 90 mg/dl and CSF protein of 375 mg/dl. Adenosine deaminase and CSF cartridge-based nucleic acid amplification test (CBNAAT) were unremarkable. The patient was started on intravenous (IV) antibiotics, IV antivirals, mannitol, and dexamethasone with other supportive medications. Reverse transcription-polymerase chain reaction (RT-PCR) for COVID-19 was done and was negative. The patient was tested for COVID-19 IgM and IgG antibodies and was positive for IgG antibodies. The patient was also tested for other causes of encephalitis; PCR test for herpes simplex virus (HSV), dengue, scrub, and leptospira was done, which were ruled out. CSF electrophoresis was done for the presence of oligoclonal bands and was negative. The blood tests and diagnostic workup of the patient are provided in Table 1.

**Table 1:** Diagnostic tests

Blood Tests and Diagnostic Workup	Result	Reference Range
Hemoglobin	11.3 g%	12-15 g%
Mean corpuscular hemoglobin concentration	32.5%	31.5-34.5%
Mean corpuscular volume	72.2 fL	80-100 fL
Mean corpuscular hemoglobin	23.5 pg	27-32 pg
Total red blood cell count	4.71 millions/cu mm	3.8-4.8 millions\cu mm
Total white blood cell count	14700/cu mm	4000-10000 cu mm
Platelet count	3.10 lakhs/cu mm	1.50-4.10 lakhs/cu mm
Random blood sugar	151 mg%	30-150 mg%
Urea	18 mg/dl	15-36 mg/dl
Creatinine	0.6 mg/dl	0.52-1.04 mg/dl
Serum sodium	129 mmol/L	137-147 mmol/L
Serum potassium	4.4 mmol/L	3.3-5.1 mmol/L
Serum albumin	4.8 g/dl	3.5-5.0 g/dl
Alkaline phosphatase	100 U/L	38-126 U/L
Bilirubin conjugated	0.2 mg/dl	0.0-0.3 mg/dl
Bilirubin unconjugated	0.1 mg/dl	0.0-1.1 mg/dl
Globulin	3.6 g/dl	2.4-3.5 g/dl
Serum glutamic pyruvic transaminase (SGPT)	15 U/L	<35 U/L
Serum glutamic-oxaloacetic transaminase (SGOT)	30 U/L	14-36 U/L
Total protein	8.4 g/dl	6.3-8.2 g/dl
Total bilirubin	0.3 mg/dl	0.2-1.3 mg/dl
Cerebrospinal fluid total white blood cell count	47 cells/cu mm	0-5 cells/cu mm
Cerebrospinal fluid glucose	78 mg/dl	50-75 mg/dl
Cerebrospinal fluid protein	375 mg/dl	170-550 mg/dl

**Figure 1:**
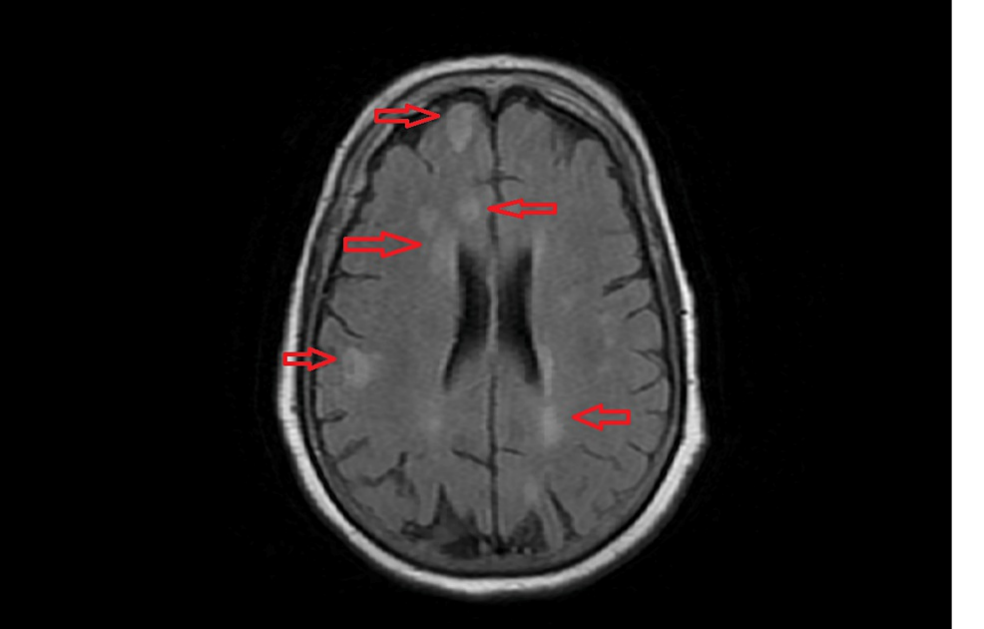
FLAIR image of the brain MRI of the patient showing multiple white matter hyperintensities suggestive of acute demyelinating disease FLAIR: fluid-attenuated inversion recovery; MRI: magnetic resonance imaging

With no improvement in the general condition of the patient in 24 hours, the patient was planned for brain magnetic resonance imaging (MRI). Brain MRI of the patient was done, which revealed the presence of multiple white matter hyperintensities in supratentorial brain parenchyma, acute demyelinating disease (Figure 1). The patient was started on pulse therapy of 1 g methylprednisolone for five days.

Outcome and follow-up

Despite a prompt diagnosis of acute disseminated encephalomyelitis (ADEM) and early initiation of the treatment, the condition of the patient did not improve. The patient could not be weaned off from the ventilator, and the patient has succumbed to the disease.

## Discussion

Transverse myelitis following vaccine administration has been reported with older vaccines, but recent studies have also shown a myelitis picture after the administration of the COVID-19 vaccine. Here, we presented a case of a female showing an acute disseminated encephalomyelitis-like picture after taking her AstraZeneca vaccine for the COVID-19 virus. There have been several mechanisms by which vaccines have been known to show transverse myelitis-like pictures. They include molecular mimicry, epitope spreading, and polyclonal activation of B lymphocytes [2]. Some vaccines have also certain ingredients such as adjuvants and preservatives that can also cause an immune response [3]. Magnetic resonance imaging is the modality of choice to diagnose cases of acute disseminated encephalomyelitis. CT imaging may not reveal the changes in ADEM. In MRI, T2-weighted imaging and fluid-attenuated inversion recovery (FLAIR) imaging can show patchy areas of increased signaling [4]. The lesions of ADEM are seen mostly in the cortical and subcortical areas of the brain and also in the brain stem and the spinal cord [5]. The common differentials of ADEM include the first attack of multiple sclerosis (MS) and neuromyelitis optica. ADEM and MS are two clinical disorders with overlapping features. However, cases of the first clinical episode of CNS demyelination showing both features of ADEM and MS do exist, suggesting that CNS pathology of ADEM may share common pathologic mechanism(s) with certain subgroups of MS [6]. ADEM has been reported with almost every vaccine available for the COVID-19 virus, namely, AstraZeneca, Pfizer, Sinovac, Janssen, and Moderna. Although most of the post-vaccination reactions are mild, there have been rare instances of life-threatening complications; neuroinflammation has also been reported [7]. Most post-mortem findings of cases of sudden deaths following vaccination have revealed the presence of extensive demyelination in the white matter. Post-mortem autopsies have revealed the cause of death in cases of post-vaccination deaths as “acute disseminated encephalomyelitis (ADEM) in the setting of recent AstraZeneca COVID-19 vaccination” [7]. New diagnostic criteria were proposed by Graus et al., which included (I) subacute onset of altered mental status or memory deficits or psychiatric symptoms (less than three months), (II) CSF pleocytosis or MRI findings suggestive of encephalitis, and (III) exclusion of alternative causes [8]. The present case has fit into the proposed criteria for autoimmune encephalitis after vaccine administration. Post-vaccination patients can also present thrombotic thrombocytopenia and myocardial infarction [9,10].

Management

The management of acute disseminated encephalomyelitis includes the administration of a high dose of steroids, and if no desired response is seen, then the administration of intravenous immunoglobulins (IVIG) is indicated. High-dose intravenous methylprednisolone of up to 20-30 mg/kg/day with a maximum dose of 1000 mg/day helps negate the immune response of the central nervous system and helps achieve clinical improvement [11]. Plasmapheresis is also indicated in severe cases [12]. There have been reports of clinical improvement in 80% of cases of diagnosed ADEM.

## Conclusions

Myelitis- and encephalitis-like characteristics after vaccination have been a common entity that has been encountered by clinicians. With the advent of newer vaccines for the SARS-CoV-2 virus and with the lack of long-term studies and the side effects of the vaccination, there have been newer symptoms up-and-coming with each passing day. For a physician encountering patients with sudden neurodeficits, the history of recent vaccinations always plays a key in the diagnosis and the early treatment of the disease. Although ADEM has been seen with good results, there have also been reported mortalities. In our case, especially, there was a late presentation of the patient 15 days after the onset of symptoms, and also contributing to her prognosis was her elderly age. ADEM, which is seen after vaccination itself, is a rare entity, and although a favorable prognosis is seen, our patient has been a rare instance of mortality due to the same, and hence, we wanted to report the case. Steroids have been beneficial in treating the disease; there are also reports of intravenous immunoglobulins with proven efficacy, but we have no reports of delayed presentations of the disease like our case has and if IVIG is beneficial in such cases. Some studies that have reported a better outcome of ADEM have also shown improvement in the lesions in the brain, and repeat CSF studies also show reduced pleocytosis. We would like to conclude by emphasizing the importance of vaccination history and prompt diagnosis of ADEM by early imaging and CSF analysis to treat the patient.
